# Associations of Prolonged Occupational Sitting with the Spectrum of Kidney Disease: Results from a Cohort of a Half-Million Asian Adults

**DOI:** 10.1186/s40798-022-00542-8

**Published:** 2022-12-13

**Authors:** Min-Kuang Tsai, Wayne Gao, Kuo-Liong Chien, Chin-Kun Baw, Chih-Cheng Hsu, Chi-Pang Wen

**Affiliations:** 1 Institute of Epidemiology and Preventive Medicine, College of Public Health, National Taiwan University, No. 17, Xu-Zhou Road, Taipei, 10055 Taiwan; 2 PhD Program in Global Health and Health Security, College of Public Health, Taipei Medical University, Taipei, Taiwan; 3 The Southeast Permanente Medical Group, Atlanta, Georgia USA; 4 Institute of Population Health Sciences, National Health Research Institutes, No. 35, Keyan Road, Zhunan Town, Miaoli, 35053 Taiwan; 5 China Medical University Hospital, Taichung, Taiwan

**Keywords:** Prolonged occupational sitting, Physical activity, Chronic kidney disease, End-stage renal disease, Cohort

## Abstract

**Background:**

Kidney diseases are viewed as continuously progressing diseases from microalbuminuria and chronic kidney disease (CKD), to end-stage renal disease (ESRD) and its mortality including deaths. The report on the association between prolonged sitting and kidney diseases is limited.

**Methods:**

We examined a cohort of 455,506 participants in a screening program in Taiwan conducted between 1996 and 2017. Data on occupational sedentary behavior and physical activity were collected with a standardized questionnaire. The outcomes of ESRD and death were identified by linking with the Catastrophic Illness Dataset and Cause of Death Data. The association between prolonged sitting and CKD, the incidence of ESRD, and death were assessed using logistic regression models to compute odds ratios (ORs) and Cox proportional hazards models for hazard ratios (HRs).

**Results:**

More than half of the participants, i.e., 265,948 (58.4%), were categorized as “prolonged sitting” during their work. During a median of 13 years of follow-up, we identified 2227 individuals undergoing dialysis and 25,671 deaths. Prolonged occupational sitting was significantly associated with a higher risk of CKD (OR: 1.26, 95% confidence interval: 1.21, 1.31), ESRD (HR: 1.19, 95% CI 1.03, 1.38), and kidney-specific mortality (HR: 1.43, 95% CI 1.07, 1.91) compared to mostly standing participants after controlling for physical activity and other risk factors. Inactive prolonged sitting carries a significantly higher risk of ESRD than physically active mostly standing participants (HR: 1.34, 95% CI 1.04, 1.73). However, active prolonged sitting decreased the risk of ESRD (HR: 1.03, 95% CI 0.79, 1.34) compared to inactive prolonged sitting.

**Conclusion:**

The results suggest that prolonged occupational sitting is associated with a greater risk of the spectrum of kidney disease, proteinuria, CKD, dialysis (ESRD), and mortality for all causes and kidney diseases. Physical activity, even at a minimal level of 15 min/day (90 min/week) of moderate-intensity exercise, was associated with a reduction in these risks.

## Key Points

- Prolonged occupational sitting was significantly associated with a higher risk of chronic kidney disease, end-stage renal disease (ESRD), and kidney-specific mortality compared to mostly standers.
- Prolonged occupational sitters had a significantly higher risk of ESRD among men, younger or older, smoking, hypertensive or diabetic participants when compared to mostly standers.
- Staying active and shortening the amount of time spent sitting may be important ways to lower the risk of the spectrum of kidney diseases.

## Introduction

In 1949, a classic study found that sedentary bus drivers who mostly sat all day had twice as many heart attacks as bus conductors who mostly stood all day [1, 2]. Since this early study, the prevalence of sedentary behavior has increased [3]. Even when physical activity guidelines are met, sitting for prolonged periods can compromise metabolic health [4]. Several studies have attempted to find an association between prolonged occupational sitting and risks of kidney disease [5–10], but the link remains uncertain. Sedentary behavior has been associated with a higher risk of proteinuria [6] which can be a sign of kidney disease, while another study found increased sedentary behavior to be associated with lower estimated glomerular filtration rate (eGFR) but not proteinuria [8]. Additionally, sitting time has been cross-sectionally associated with chronic kidney disease (CKD) [5, 8, 10] and yet there are still very limited longitudinal studies to explore if sedentary behavior is prospectively associated with a high risk of kidney diseases [11]. To understand such causal relationships, researchers need to understand the link between prolonged occupational sitting and CKD spectrum, including low eGFR, proteinuria, end-stage renal disease (ESRD), and kidney-disease-related deaths.

In 2020, the World Health Organization (WHO) addressed sedentary behavior in guidelines for physical activity and indicated prolonged sitting as a risk behavior [12]. These guidelines provided, for the first time, recommendations related to the association of sedentary behavior with health outcomes. Improving Global Kidney Disease Improving Global Outcomes in 2020 [13] also advised the avoidance of sedentary behavior, suggesting that sedentary behavior is associated with a higher risk of hospitalization due to CKD and death. These guidelines emphasize reducing sedentary behavior and increasing the amount of physical activity to reduce the risk of kidney disease. However, there is still limited evidence on whether prolonged occupational sitting increases the risk of kidney disease.

With the world’s highest ESRD prevalence in Taiwan [14], we were able to link data from a large cohort comprising more than a half-million individuals with the Catastrophic Illness Registry [15], which maintained a national record of all patients with ESRD. We hypothesized that prolonged occupational sitting would be associated with CKD and ESRD, after adjustment for physical activity and other cardiovascular confounders. The objectives of this analysis were: (1) to quantify the relationship between prolonged occupational sitting and risk of kidney disease, including CKD, ESRD, and mortality due to kidney disease; (2) to explore the interaction between prolonged occupational sitting and physical activity on ESRD risk; (3) and to investigate whether prolonged occupational sitting is associated with a higher risk of ESRD in subgroups based on age, sex, and cardiovascular disease risk factors.

## Methods

### Participants

**Fig. 1:**
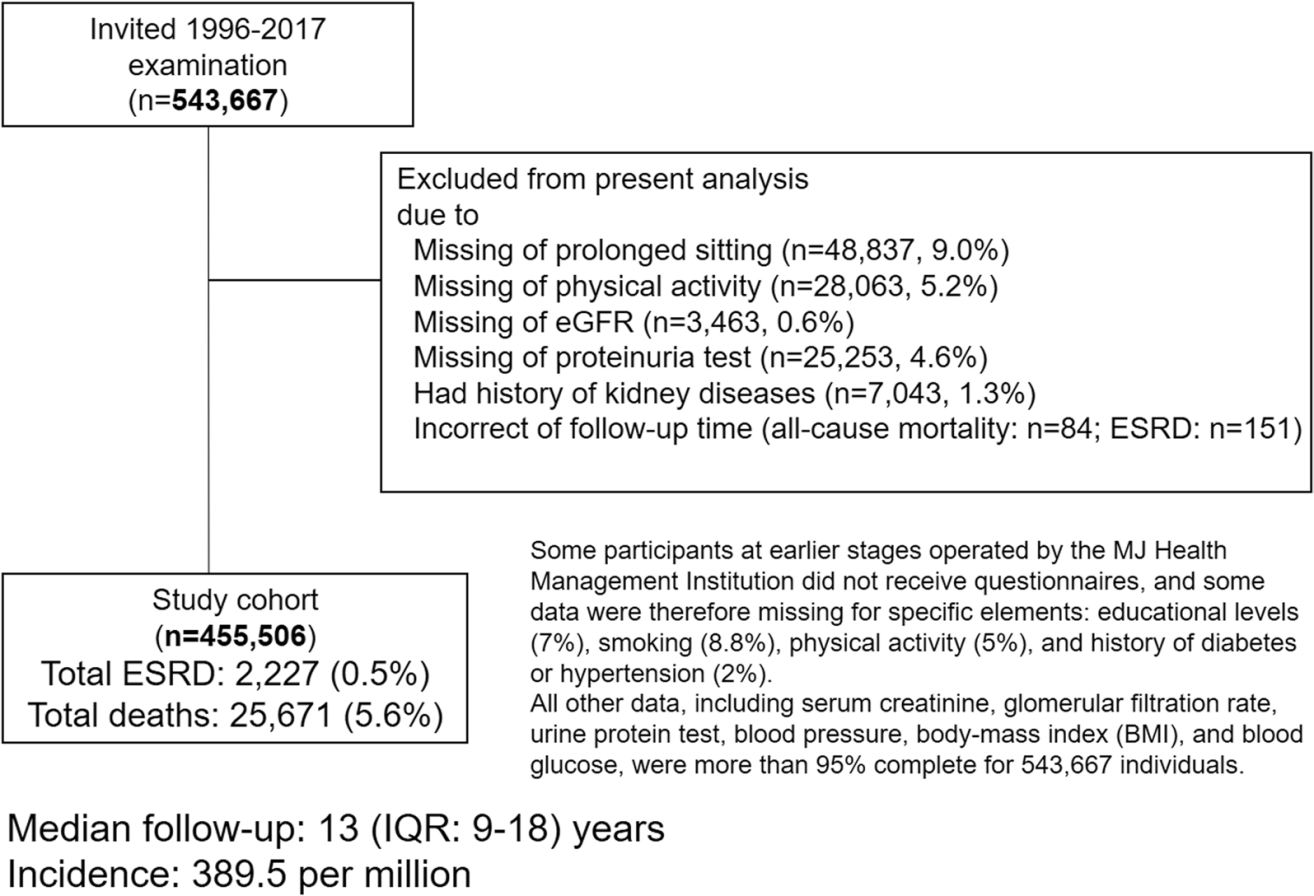
Flowchart of participants included in this study. *eGFR* Estimated glomerular filtration rate; *ESRD* End-stage renal disease; *IQR* Interquartile range

The Taiwan MJ Cohort builds upon a large database and biorepository of healthy individuals undergoing a health screening program in Taiwan conducted by MJ Group. There are four centers in the northern (Taipei), northwest (Taoyuan), central (Taichung), and southern (Kaohsiung) parts of Taiwan. These participants are from all over Taiwan. All four centers used identical screening procedures with the same models of instruments, and the results were centrally managed and stored. Participants underwent a standardized physical examination and biochemical blood and urine tests and completed a self-administered questionnaire on lifestyle and medical history. All participants were encouraged to visit on a yearly basis, and the same questionnaires were filled out on every examination, but results from the initial visit were used in this study. We excluded participants with the following conditions: missing data on prolonged occupational sitting (9.0%), physical activity (5.2%), eGFR (0.6%), or proteinuria (4.6%). Participants with a history of kidney disease (1.3%) and those undergoing dialysis before the screening (0.04%) were also excluded (see Fig. 1). We conducted our analysis in the Taiwan MJ Cohort comprising 455,506 participants aged 20 years or older who underwent a series of medical screenings between 1996 and 2017. The 21-year study period yielded 5.87 million person-years of follow-up for ESRD. This study has received ethical approval from China Medical University and was conducted according to the Declaration of Helsinki. All data related to personal identification were removed, and analyses were conducted at the Data Science Center of the Ministry of Health and Welfare in Taiwan.

### Prolonged Occupational Sitting and Physical Activity

We defined prolonged sitting status using results from the questionnaire on occupational activity. Participants were asked, ‘During your work, do you “mostly sit,” “alternate between sitting and standing,” “mostly stand” or “use all of your major muscles?”.’ We defined respondents who indicated they “mostly sit” during work as prolonged occupational sitters and those who indicated they “mostly stand” as occupational non-sitters. The reliability of occupational sitting status was measured by consistency in questionnaire answers given from two different visits. There was an acceptable correlation of occupational sitting status in participants with data for two visits (time interval: 2.2 ± 2.06 years), with a 0.63 (*p* < 0.001) Spearman’s rank correlation coefficient (Appendix Table 5). We compared ESRD outcomes in participants with at least two visits who were consistent in their reporting of occupational activity. Compared with participants in the mostly standing group, participants in the mostly sitting group had HRs for ESRD of 1.23 (95% CI 1.07, 1.40) on their first visit and 1.24 (0.96, 1.20) on their second visit. These values were sufficiently close, indicating that the validity of our questionnaire was within an acceptable range (Appendix Table 6). Participants were also asked to indicate the type and duration of weekly leisure time physical activity (LTPA). We classified participants into three categories based on their volume of PA per week. The PA categories, based on Ainsworth’s Compendium [16], are inactive (< 3.75 MET-hour), low (3.75–7.49 MET-hour), and medium or above (≥ 7.50 MET-hour). Participants in the medium or above group were in physical activity and met the recommended amount of PA for adults of at least 150 min of moderate-intensity exercise per week. The reliability and validity of the PA questionnaire have been previously demonstrated [17–19].

### Chronic Kidney Disease, End-Stage Renal Disease, and Mortality Due to Kidney Disease

We used the Chronic Kidney Disease Epidemiology Collaboration equation to estimate the glomerular filtration rate [20]. We used the Roche Miditron M semi-automated computer-assisted urinalysis system to test urinary protein, with positive results reported as trace, 1+ , 2+ or more. The definition of chronic kidney disease is as follows: Stage 1 CKD: eGFR ≥ 90 mL/min/1.73 m^2^ and the detection of proteinuria is more than trace; Stage 2 CKD: eGFR 60–89 mL/min/1.73 m^2^ and the examination of proteinuria is more than trace; Stage 3 chronic kidney disease: eGFR 30–59 mL/min/1.73 m^2^; Stage 4 chronic kidney disease: eGFR 15–29 mL/min/1.73 m^2^; and Stage 5 chronic kidney disease: eGFR < 15 mL/min/1.73 m^2^. The incidence of ESRD was ascertained by linking the identification numbers of individual cohort members with the nationwide Registry of Patients with Catastrophic Illness through the end of 2017 using International Classification of Diseases (ICD) diagnostic codes. For the study cohort, we included individuals diagnosed with ESRD based on ICD-9-CM code 585 or ICD-10-CM codes N18.5 and N18.6 as well as those with a kidney transplant [15]. Taiwan has a registry of deaths of citizens coded from death certificates. Causes of death are classified according to ICD-9 or ICD-10 codes and consist of all-cause, cardiovascular disease, diabetes, and kidney diseases.

### Covariates

Education levels were classified as middle school or lower, high school, junior college, and college or higher. Smoking status includes current, ex-smokers, and never-smokers from self-reported questionnaire. Drinking was defined as drinking alcohol three times or more a week and two or more drinks each time. Participants were defined as having diabetes, hypertension, or hyperlipidemia if they had a history of these diseases or positive screening results such as fasting blood glucose ≥ 126 mg/dL or systolic blood pressure ≥ 140 mmHg or total cholesterol ≥ 240 mg/dL, respectively. Participants were asked whether they were long-term users of Chinese herbal medicines or painkillers. Participants with a body mass index (BMI) ≥ 25 kg/m^2^ and < 30 kg/m^2^ were classified as overweight. Participants with a BMI ≥ 30 kg/m^2^ were classified as obese.

### Statistical Analysis

We calculated odds ratios (ORs) to compare the risk of kidney disease, including CKD, eGFR < 60 mL/min/1.73 m^2^, and proteinuria (trace or above) for prolonged occupational sitters, those who alternate between sitting and standing, and those who reported “using all of your major muscles compared to mostly standers. We calculated hazard ratios (HRs) to compare ESRD and mortality risk among the occupational activity categories (i.e., prolonged sitters, alternating sitting and standing, and non-sitters). Categorical variables were sex, education; smoking; alcohol drinking; diagnosis of diabetes, hypertension, or hyperlipidemia; long-term use of herbal medicine; long-term use of painkillers; and physical activity levels. Continuous variables were age and BMI. Time at entry was the date of recruitment, and time of exit was the end of follow-up (Dec 31, 2017), or date of death, or date of ESRD diagnosis, whichever was earlier. We calculated HRs using multivariate Cox proportional hazards models and adjusted for age, sex, education level, smoking status, drinking status, BMI, diabetes status, hypertension status, long-term use of herbal medicine, long-term use of painkillers, and physical activity. We conducted stratified analyses to evaluate a potential effect modification by baseline sex, age (60 years as cutoff), and other covariates, and no significant interaction was found. In addition, we analyzed the incidence of CKD in participants who had two visits. About half (205,617 (45%)) had data for two visits. We excluded those with CKD at the first visit, including 14,512 people with CKD stages 1 to 5, as well as those with proteinuria (*n* = 10,311) (Appendix Fig. 3). Data were available for two visits for 174,366 people who did not have CKD at the first visit. We analyzed these data to determine if prolonged occupational sitters had a higher risk of developing CKD, proteinuria, or eGFR < 60 mL/min/1.73 m^2^ at the second visit than those who were mostly standers. We performed sensitivity analyses by excluding participants undergoing dialysis or with a kidney transplant within the first 2 years of follow-up or those with stage 5 CKD (eGFR < 15 mL/min/1.73 m^2^). All statistical analyses were performed in SAS version 9.4. Two-sided *p* < 0.05 was considered statistically significant.

## Results

Table 1 presents the characteristics of 455,506 participants in the four prolonged occupational sitting categories. More than half of the participants (58.4%) were prolonged occupational sitters, and one-tenth (10.5%) were mostly standers. Compared to mostly standers, prolonged occupational sitters tended to be younger, female, inactive and have a higher education level (see Table 1). Prolonged sitters had lower rates of proteinuria (5.9% vs. 6.9%), although eGFR < 60 mL/min/1.73 m^2^ was similar for both groups (3.8% vs. 3.8%). While the group of prolonged occupational sitters included fewer individuals with CKD (8.9% vs. 10.0%), ORs adjusted for age and covariates indicated that prolonged occupational sitters had a higher risk of CKD (OR: 1.26, 95% CI 1.21, 1.31), proteinuria (OR: 1.12, 95% CI 1.08, 1.18), and eGFR < 60 (OR: 1.46, 95% CI 1.38, 1.56) compared to mostly standers (see Tables 2 and 3).

**Table 1:** Characteristics of participants by occupational sedentary status

	Total		Mostly sitting		Often alternating sitting and standing		Mostly standing		Using all of your major muscles	
	*N*	(%)	*N*	(%)	*N*	(%)	*N*	(%)	*N*	(%)
Total	455,506	(100.0)	265,948	(58.4)	129,035	(28.3)	48,016	(10.5)	12,507	(2.7)
Age (year)										
20–39	268,090	(58.9)	162,544	(61.1)	74,897	(58.0)	24,140	(50.3)	6509	(52.0)
40–59	141,074	(31.0)	77,238	(29.0)	41,171	(31.9)	17,844	(37.2)	4821	(38.5)
≥ 60	46,342	(10.2)	26,166	(9.8)	12,967	(10.0)	6032	(12.6)	1177	(9.4)
Sex										
Men	228,913	(50.3)	124,217	(46.7)	62,126	(48.1)	31,846	(66.3)	10,724	(85.7)
Women	226,593	(49.7)	141,731	(53.3)	66,909	(51.9)	16,170	(33.7)	1783	(14.3)
Education										
Middle school or below	92,294	(20.5)	35,338	(13.5)	33,539	(26.4)	18,112	(38.5)	5305	(43.3)
High school	95,250	(21.2)	42,045	(16.0)	35,745	(28.1)	12,971	(27.5)	4489	(36.6)
Junior college	92,721	(20.6)	55,443	(21.1)	28,208	(22.2)	7588	(16.1)	1482	(12.1)
College or above	168,990	(37.6)	129,850	(49.4)	29,732	(23.4)	8427	(17.9)	981	(8.0)
Smoking status										
Non-smoker	313,468	(70.8)	196,932	(75.9)	85,110	(68.3)	26,359	(56.9)	5067	(41.8)
Ex-smoker	27,956	(6.3)	15,223	(5.9)	7535	(6.0)	3991	(8.6)	1207	(10.0)
Current smoker	101,202	(22.9)	47,424	(18.3)	31,921	(25.6)	16,015	(34.5)	5842	(48.2)
Drinking status										
Non-drinker	349,205	(80.1)	214,758	(83.8)	96,040	(78.3)	31,508	(69.4)	6899	(58.0)
Occasional drinker	49,323	(11.3)	24,878	(9.7)	15,057	(12.3)	7077	(15.6)	2311	(19.4)
Regular drinker	37,638	(8.6)	16,635	(6.5)	11,512	(9.4)	6801	(15.0)	2690	(22.6)
BMI (kg/m^2^)										
< 18.5	38,441	(8.4)	24,464	(9.2)	10,233	(7.9)	3153	(6.6)	591	(4.7)
18.5–24.9	291,131	(63.9)	171,603	(64.5)	81,743	(63.4)	30,198	(62.9)	7587	(60.7)
25–29.9	106,094	(23.3)	58,729	(22.1)	31,256	(24.2)	12,422	(25.9)	3687	(29.5)
≥ 30	19,718	(4.3)	11,057	(4.2)	5784	(4.5)	2236	(4.7)	641	(5.1)
Hypertension										
No	374,557	(82.2)	221,436	(83.3)	105,172	(81.5)	38,108	(79.4)	9841	(78.7)
Yes	80,949	(17.8)	44,512	(16.7)	23,863	(18.5)	9908	(20.6)	2666	(21.3)
Diabetes										
No	433,111	(95.1)	253,040	(95.1)	122,805	(95.2)	45,443	(94.6)	11,823	(94.5)
Yes	22,395	(4.9)	12,908	(4.9)	6230	(4.8)	2573	(5.4)	684	(5.5)
Hyperlipidemia										
No	402,866	(88.5)	235,668	(88.6)	113,907	(88.3)	42,216	(88.0)	11075	(88.6)
Yes	52,519	(11.5)	30,236	(11.4)	15,087	(11.7)	5,770	(12.0)	1426	(11.4)
Painkiller use										
No	441,053	(97.9)	258,794	(98.2)	124,724	(97.6)	45,520	(97.2)	12,015	(96.9)
Yes	9425	(2.1)	4631	(1.8)	3081	(2.4)	1327	(2.8)	386	(3.1)
Chinese herbal medicine use										
No	417,779	(91.9)	245,176	(92.4)	118,073	(91.7)	43,400	(90.9)	11,130	(89.1)
Yes	36,583	(8.1)	20,179	(7.6)	10,670	(8.3)	4368	(9.1)	1366	(10.9)
Physical activity										
Inactive	225,410	(49.5)	124,624	(46.9)	66,060	(51.2)	27,272	(56.8)	7454	(59.6)
Low	120,192	(26.4)	76,418	(28.7)	32,390	(25.1)	9428	(19.6)	1956	(15.6)
Medium	68,455	(15.0)	42,234	(15.9)	18,905	(14.7)	5921	(12.3)	1395	(11.2)
High	25,938	(5.7)	14,990	(5.6)	7375	(5.7)	2852	(5.9)	721	(5.8)
Very high	15,511	(3.4)	7682	(2.9)	4305	(3.3)	2543	(5.3)	981	(7.8)
Proteinuria										
Negative	427,343	(93.8)	250,365	(94.1)	120,707	(93.5)	44,699	(93.1)	11,572	(92.5)
Trace	22,391	(4.9)	12,222	(4.6)	6784	(5.3)	2654	(5.5)	731	(5.8)
1+	3587	(0.8)	2107	(0.8)	961	(0.7)	397	(0.8)	122	(1.0)
2+	1387	(0.3)	812	(0.3)	351	(0.3)	179	(0.4)	45	(0.4)
3+ or more	798	(0.2)	442	(0.2)	232	(0.2)	87	(0.2)	37	(0.3)
eGFR (mL/min/1.73 m^2^)										
> 60	438,899	(96.4)	255,718	(96.2)	124,942	(96.8)	46,173	(96.2)	12,066	(96.5)
≤ 60	16,607	(3.6)	10,230	(3.8)	4093	(3.2)	1843	(3.8)	441	(3.5)
CKD										
None-CKD	414,139	(90.9)	242,286	(91.1)	117,419	(91.0)	43,213	(90.0)	11,221	(89.7)
Stage 1	10,447	(2.3)	5639	(2.1)	3329	(2.6)	1140	(2.4)	339	(2.7)
Stage 2	14,313	(3.1)	7793	(2.9)	4194	(3.3)	1820	(3.8)	506	(4.0)
Stage 3–5	16,607	(3.6)	10,230	(3.8)	4093	(3.2)	1843	(3.8)	441	(3.5)

*N* Number of participants; *BMI* Body mass index; *eGFR* Estimated glomerular filtration rate; *CKD* Chronic kidney disease

**Table 2:** Risk of kidney diseases including CKD (proteinuria and lower eGFR) by occupational sedentary status from cross-sectional data

	Mostly sitting			Often alternating sitting and standing			Mostly standing		Using all of your major muscles		
	*n* of diseases	OR	(95% CI)	*n* of diseases	OR	(95% CI)	*n* of diseases	OR	*n* of diseases	OR	(95% CI)
CKD	23,662	1.26	(1.21, 1.31)	11,616	1.10	(1.05, 1.14)	4803	1	1286	0.98	(0.91, 1.05)
Proteinuria	15,583	1.12	(1.08, 1.18)	8328	1.04	(1.00, 1.09)	3317	1	935	0.97	(0.90, 1.06)
eGFR < 60 (mL/min/1.73 m^2^)	10,230	1.46	(1.38, 1.56)	4093	1.19	(1.12, 1.28)	1843	1	441	1.02	(0.90, 1.15)

**Table 3:** Risk of ESRD and mortality due to kidney disease by occupational sedentary status from longitudinal follow-up data

	*n* of ESRD	HR	(95% CI)	*n* of ESRD	HR	(95% CI)	*n* of ESRD	HR	*n* of ESRD	HR	(95% CI)
ESRD	1217	1.19	(1.03, 1.38)	640	1.05	(0.90, 1.22)	285	1	85	1.11	(0.86, 1.43)

	*n* of mortality	HR	(95% CI)	*n* of mortality	HR	(95% CI)	*n* of mortality	HR	*n* of mortality	HR	(95% CI)
All-cause mortality	14,067	1.10	(1.05, 1.14)	6851	0.99	(0.95, 1.03)	3777	1	976	1.01	(0.94, 1.08)
CVD mortality	3064	1.27	(1.16, 1.39)	1333	1.08	(0.98, 1.20)	695	1	196	1.15	(0.98, 1.37)
DM mortality	905	1.47	(1.21, 1.78)	344	1.10	(0.90, 1.36)	151	1	42	1.16	(0.81, 1.65)
Kidney mortality	392	1.43	(1.07, 1.91)	132	0.98	(0.71, 1.34)	67	1	19	1.17	(0.68, 2.00)

Odds ratios were adjusted for age, sex, education, smoking status, drinking status, BMI, diabetes, hypertension, hyperlipidemia, long-term use of herbal medicine, long-term use of painkillers, and physical activity

Hazard ratios were adjusted for age, sex, education, smoking status, drinking status, BMI, diabetes, hypertension, hyperlipidemia, long-term use of herbal medicine, long-term use of painkillers, and physical activity

*OR* Odds ratio; *CI* Confidence interval; *HR* Hazard ratio; *CKD* Chronic kidney disease; *eGFR* Estimated Glomerular filtration rate; *ESRD* End-stage renal disease; *CVD* Cardiovascular disease; *DM* Diabetes mellitus; *BMI* Body mass index

During the median 13-year follow-up, we identified 2227 individuals undergoing dialysis and 25,671 deaths. Prolonged occupational sitters had a significantly higher risk of ESRD (HR: 1.19, 95% CI 1.03, 1.38) compared to mostly standers adjusted for physical activity and other confounders. After adjusting for the same confounders as the previous results (Tables 2 and 3), prolonged occupational sitters had a 43% increased risk of mortality due to kidney disease compared to mostly standers (HR: 1.43, 95% CI 1.07, 1.91). Prolonged sitters also had an increased risk of all-cause (HR: 1.10, 95% CI 1.05, 1.14), cardiovascular disease (CVD) (HR: 1.27, 95% CI 1.16, 1.39), and diabetes (HR: 1.47, 95% CI 1.21, 1.78) mortality. We analyzed the incidence of CKD in participants who had two visits (Appendix Table 7). Data on two visits were available for 174,366 people who did not have CKD on the first visit. Of these people, 8505 developed CKD by the second visit; 6131 had proteinuria by the second visit; and 2587 had eGFR < 60 mL/min/1.73 m^2^ at the second visit. Prolonged occupational sitters did not have a significantly increased risk of developing CKD (HR: 1.02, 95% CI 0.95, 1.10) and developing proteinuria (HR: 0.94, 95% CI 0.86, 1.10) compared to mostly standers. However, prolonged occupational sitters had a 25% increased risk compared to mostly standers for eGFR < 60 mL/min/1.73 m^2^ (Appendix Table 8).

**Fig. 2:**
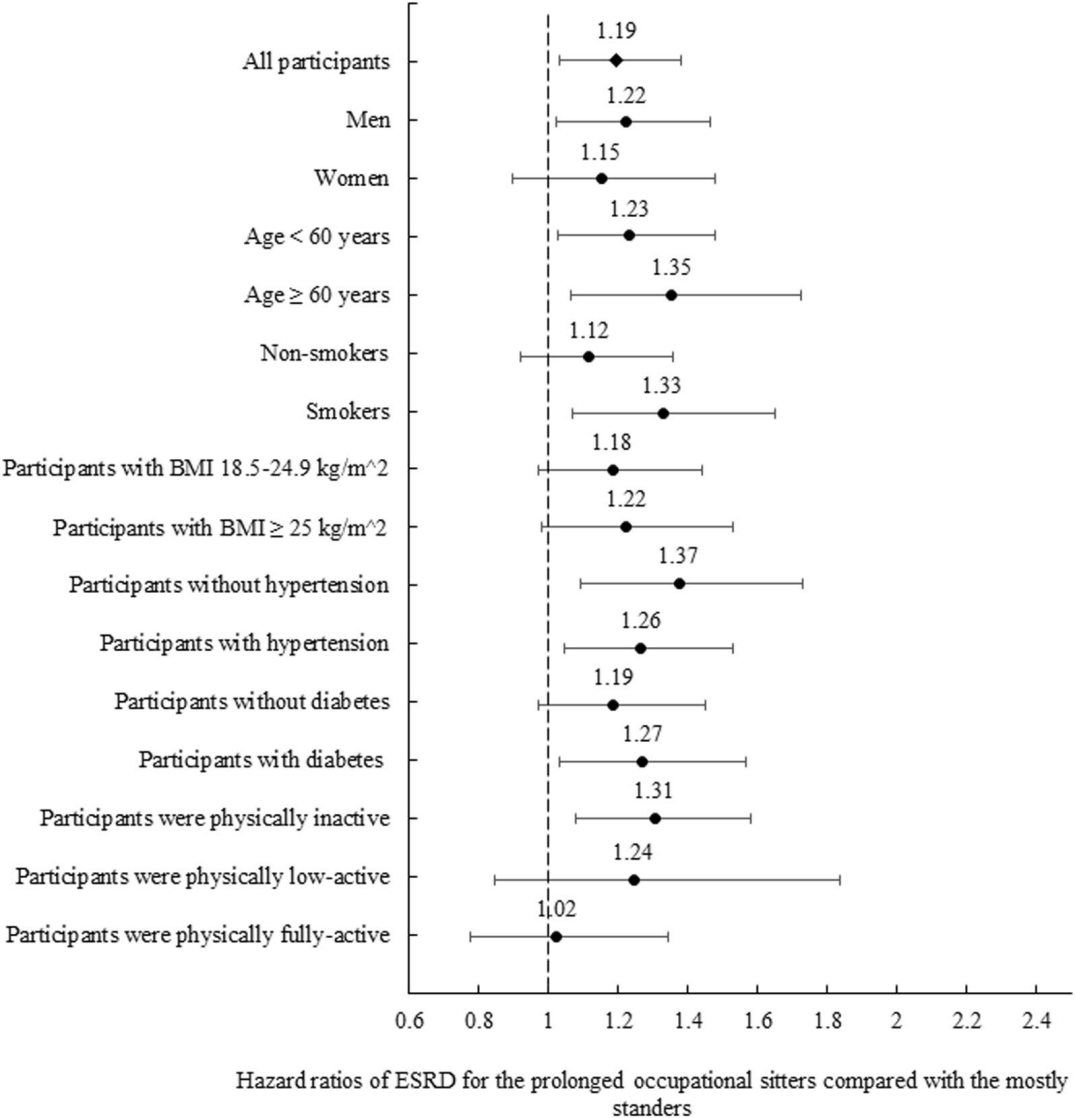
Adjusted ESRD hazard ratios for the prolonged occupational sitters compared with the mostly standers, by age, sex, smoking, body mass index (BMI), hypertension, diabetes, and physical activity amounts. *ESRD* End-stage renal disease

We conducted sensitivity analyses by excluding participants undergoing dialysis or with a kidney transplant within the first 2 years of follow-up, prolonged occupational sitters still had a significantly higher risk of ESRD (HR: 1.17, 95% CI 1.01, 1.36) compared to mostly standers (Appendix Table 9). When excluding individuals with stage 5 CKD at baseline, the results were similar (HR: 1.19, 95% CI 1.03, 1.38). In stratification analysis, there was no significant interaction between each covariate and prolonged sitting of the risk of ESRD. We found that prolonged sitters had a significantly higher risk of ESRD among male, younger or older, smoking, hypertensive or diabetic participants when compared to mostly standers (Fig. 2). Prolonged occupational sitting participants who were women, non-smoking, BMI: 18.5–24.9 kg/m^2^, overweight, non-diabetes and low or fully active had a lower risk of ESRD compared to mostly standing participants although this was not statistically significant.

Comparing the joint effects of prolonged sitting and physical inactivity on ESRD risk, inactive prolonged sitters had a 34% increased risk of ESRD compared to active mostly standers (HR: 1.34, 95% CI 1.04, 1.73). However, the risk decreased for low-active or fully active prolonged sitters (low-active prolonged sitters: HR: 1.20, 95% CI 0.91, 1.58; fully active prolonged sitters: HR: 1.03, 95% CI 0.79, 1.34) (Table 4).

**Table 4:** A joint effect of occupational sedentary status and physical inactivity on risk of ESRD

	Mostly sitting			Often alternating sitting and standing			Mostly standing			Using all of your major muscles		
Physical activity	*n* of ESRD	HR	(95% CI)	*n* of ESRD	HR	95% CI	*n* of ESRD	HR	95% CI	*n* of ESRD	HR	95% CI
Inactive	588	1.34	(1.04, 1.73)	310	1.07	(0.81, 1.40)	178	1.06	(0.79, 1.41)	64	1.34	(0.94, 1.91)
Low	250	1.20	(0.91, 1.58)	146	1.15	(0.86, 1.55)	36	0.90	(0.59, 1.38)	5	0.68	(0.27, 1.68)
Medium or above	379	1.03	(0.79, 1.34)	184	0.98	(0.74, 1.30)	71	1.00		16	0.83	(0.48, 1.46)

Hazard ratios were adjusted for age, sex, education, smoking status, drinking status, body mass index, diabetes, hypertension, hyperlipidemia, long-term use of herbal medicine, and long-term use of painkillers

*ESRD* End-stage renal disease; *HR* Hazard ratio; *CI* Confidence interval

## Discussions

The results of this study indicate the association of prolonged occupational sitting with the spectrum of kidney disease, including CKD, ESRD, and mortality due to kidney disease. Compared with mostly standers, prolonged occupational sitters had a higher risk of proteinuria (trace or above), CKD, and low eGFR. Furthermore, prolonged occupational sitters had a higher risk of ESRD, kidney-disease-related mortality, cardiovascular disease mortality, and diabetes mortality compared with mostly standers. Prolonged occupational sitting was consistently associated with a higher risk of dialysis in smokers, hypertensive and diabetic participants. The joint-effect analysis showed that inactive prolonged sitters had an even higher risk of ESRD compared with active mostly standers. In addition, greater physical activity was associated with a significantly lower kidney risk.

The results show that prolonged occupational sitting is associated with the spectrum of kidney diseases, especially for the development of ESRD and kidney-disease-related mortality. Our results are comparable with those of previous studies. A study conducted in Japan explored the amount of time elderly people spend being sedentary and the risk of lower eGFR [7]. Another study from Japan, also found that a 60 min increase in sedentary behavior, measured by an accelerometer, was associated with lower eGFR (< 60 versus ≥ 60 mL/min/1.73 m^2^) in women [21]. Elderly individuals who were sedentary and sat for more than 8 hours per day had a higher rate of low eGFR than those who sat for less than 4 hours per day (OR: 1.42, 95% CI 1.02, 1.37) [7]. The risk was similar for the mostly sitting group in the present study (OR: 1.46, 95% CI 1.38, 1.56). Another study, also conducted in Japan but focused on middle-age individuals (20–64 years old), found that for male participants, occupational sedentary behavior was related to proteinuria (1+ or more) (OR: 1.35, 95% CI 1.11, 1.63) [6]. Males in our study demonstrated a similar risk of proteinuria (OR 1.22, 95% CI 1.02, 1.46). However, the definition of proteinuria in our study is trace proteinuria or above, which is similar to microalbuminuria (albuminuria–creatinine ratio 10–29 mg/g) [22]. This means prolonged occupational sitting was associated with early stages of kidney damage. We found that prolonged occupational sitting resulted in a slightly higher risk of low eGFR (OR: 1.46) than proteinuria (OR: 1.12). The analysis of those with two visits revealed a higher risk of lower eGFR (< 60 mL/min/1.73 m^2^) for prolonged occupational sitters compared to mostly standers (HR: 1.25). However, there was no difference in the risk of proteinuria for prolonged occupational sitters compared to mostly standers (HR: 0.94). Other studies have suggested that sedentary behavior is associated with lower eGFR [10] but not proteinuria [8].

Some studies have explored the relationship between sedentary behavior and CKD [5, 8, 10]. We also found that after adjustment for physical activity, prolonged occupational sitters showed an increased risk of CKD. We found the risk of CKD for prolonged sitters was 26% higher than mostly standers after controlling for different amounts of physical activity. In order to better confirm the causality of prolonged sitting for CKD, we included the outcomes for ESRD and kidney-related deaths. Because CKD patients may die from diseases other than kidney disease [23], such as cardiovascular disease or diabetes [24], we also included these causes of death. We found that prolonged sitters had a higher risk of ESRD and kidney mortality, CVD mortality, diabetes mortality, and all-cause mortality compared with mostly standers. Prior work has shown that individuals with kidney disease spend longer sitting than healthy individuals [25]. To avoid reverse causality [26], we conducted sensitivity analyses by excluding individuals undergoing dialysis within 2 years of follow-up and those with kidney disease (Appendix Table 8). These analyses support a causal relationship between sedentary behavior and kidney disease.

In this study, the ESRD risk for prolonged occupational sitters could be independent of physical activity. Both prolonged sitting and physical inactivity significantly increased the risk of ESRD. Inactive prolonged sitters had a 34% higher risk of ESRD than active non-sitters. Moreover, the risk decreased for prolonged occupational sitters if they were physically active. Prior studies have suggested that sedentary behavior and physical inactivity are two separate risk factors for all-cause and CVD mortality [27]. In the current study, both sedentary behaviors and physical inactivity increased the risk for CKD, ESRD, and mortality due to kidney disease. In the stratified analyses of several cardiovascular factors including smokers, hypertensive, diabetic, or participants who are overweight, prolonged sitting was consistently associated with a higher risk of dialysis. However, women had a relatively lower risk than men. Prior work has found that women have a lower risk from prolonged sitting than men and that this may be due to potential confounders [28]. It has been found that men store a greater proportion of total body fat as visceral fat, which is more associated with risks of chronic disease than gluteofemoral obesity [28]. However, some studies found that women are more at risk for developing the disease due to sedentary behavior [5, 29]. The gender difference for the ESRD risk of prolonged occupational sitting needs to be further investigated.

We also found that the ESRD risk for prolonged occupational sitters in participants who are overweight was marginally higher compared to the risk for mostly standers (HR: 1.22, 95% CI 0.98, 1.53). It is probably due to the risk of dialysis increased among participants who are overweight, and it may be affected by other confounders that are associated with being overweight [30]. Previous studies have suggested that prolonged sitters are at higher risks of obesity and diabetes [31–33]. Individuals who are overweight are more likely to have cardio-metabolic risk biomarkers [34]. In this cohort, we found that the risk of death due to diabetes was higher for sitters than non-sitters (Table 3) and that sitters were at greater risk for diabetes and being overweight (OR: 1.26, 95% CI 1.20, 1.32 for diabetes; OR: 1.15, 95% CI 1.12, 1.18 for overweight, Appendix Table 9). The risk of obesity and diabetes may play an important role in prolonged occupational sitters’ risk of ESRD and mortality due to kidney disease. The underlying reasons that sedentary behavior is harmful may be related to diseases such as cardiovascular disease [31, 35, 36]. In addition, it is well known that sedentary individuals tend to have increased blood level of neutral fat and decreased high-density cholesterol [37]. A 2012 study conducted in Australia found that sitting and watching TV after a meal results in higher blood sugar than standing or walking for 20 min [38]. The results of prior work suggested that engaging in one hour of exercise per day offsets the harm caused by a sedentary lifestyle [39]. We found that participants who were prolonged occupational sitters but met recommended physical activity guidelines or engaged in at least 90 min of moderate-intensity exercise per week (15 min per day) had an even lower risk of ESRD compared to physically inactive prolonged sitters.

### Strengths and Limitations

This study has several strengths. First, with more than 20 years of follow-up and a large cohort, there were sufficient ESRD cases and mortality due to kidney disease to test the effect of prolonged occupational sitting. Second, the availability of data for confounders’ information such as physical activity allowed us to show the independent effects of prolonged sitting [17]. Third, sensitivity analyses that excluded those undergoing dialysis within 2 years allowed us to clarify the causal relationship between prolonged occupational sitting and kidney disease. Fourth, we were able to examine the whole spectrum of kidney disease for prolonged sitters by cross-sectional and longitudinal outcomes. Lastly, this is one of only a few studies to investigate the association of lifestyle risk factors such as prolonged occupational sitting and physical activity with the risk of dialysis, which is important due to the growing incidence of dialysis globally and in Taiwan. Despite its strengths, this study had some limitations. First, prolonged occupational sitting and physical activity were self-reported and some recall bias could exist. However, many disease and mortality outcomes in this cohort have been shown to have a dose–response relationship with physical activity [17, 19]. Second, prolonged occupational sitting is derived from self-reported questionnaire in this study as occupational sedentary behavior, with four categorical sitting status at work instead of continuous data measured by an objective accelerometer. Third, the studied population was Asian from a self-paid health examination enterprise, and participants are likely to have a higher socioeconomic status than the general public.

## Conclusion

In summary, after adjustment for physical activity, prolonged occupational sitting was associated with a higher risk of CKD, ESRD, and mortality due to kidney disease. Inactive prolonged sitters had a higher risk of ESRD than physically active, mostly standers. However, active prolonged sitters had decreased risk of ESRD compared to inactive prolonged sitters. During the COVID-19 pandemic, because of recommendations to stay home, the amount of time people spent time sitting increased by 28% [40]. The results of this study may be used to inform inactive prolonged sitters who work at home to engage in at least some physical activity (a minimal level of 90 min/week of moderate-intensity exercise) after spending an extended period of time sitting. Staying active and shortening the amount of time spent sitting may be important ways to lower the risk of the spectrum of kidney diseases.

## Figures and Tables

**Fig. 3:**
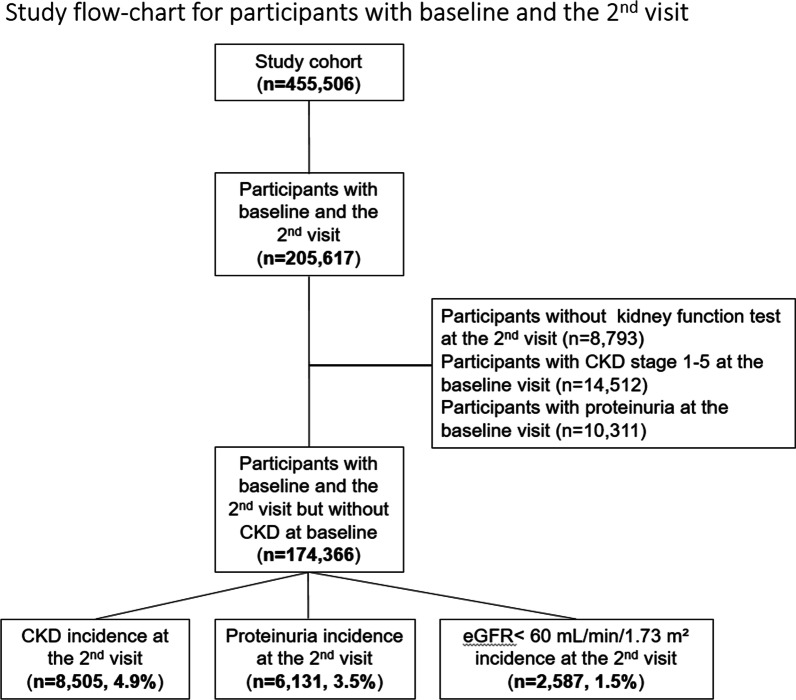
Flowchart of participants with baseline (first visit) and the second visit

## Data Availability

The Taiwan MJ Cohort is available to the worldwide research community and offers opportunities for collaboration. Those applying for data access should contact the MJ Health Research Foundation at http://www.mjhrf.org/.

## Appendix

See Tables 5, 6, 7, 8 and 9; Fig. 3.

**Table 5:** Distribution of occupational sedentary status for the participants with two visits

	Baseline data (1st visit)									
2nd visit	Mostly sitting		Often alternating sitting and standing		Mostly standing		Using all of your major muscles		Total	
	*N*	(%)	*N*	(%)	*N*	(%)	*N*	(%)	*N*	(%)
Mostly sitting	106,994	(52.0)	18,439	(9.0)	2778	(1.4)	541	(0.3)	128,752	(62.6)
Often alternating sitting and standing	14,708	(7.2)	32,515	(15.8)	6101	(3.0)	1113	(0.5)	54,437	(26.5)
Mostly standing	2228	(1.1)	5632	(2.7)	9270	(4.5)	1173	(0.6)	18,303	(8.9)
Using all of your major muscles	341	(0.2)	873	(0.4)	1006	(0.5)	1905	(0.9)	4125	(2.0)
Total	124,271	(60.4)	57,459	(27.9)	19,155	(9.3)	4732	(2.3)	205,617	(100.0)

The Spearman’s rank correlation coefficient of prolonged occupational sitting for the participants with two visits was 0.63 (*p* < 0.001)

**Table 6:** ESRD risk by occupational sedentary status for the participants with two visits

	*N*	*n* of ESRD	HR	(95% CI)
Baseline with 2nd visit				
Mostly sitting	124,271	411	1.23	(1.07, 1.40)
Often alternating sitting and standing	57,459	247	1.04	(0.90, 1.20)
Mostly standing	19,155	89	1.00	
Using all of your major muscles	4732	24	1.09	(0.86, 1.39)
2nd visit				
Mostly sitting	128,752	437	1.24	(0.96, 1.60)
Often alternating sitting and standing	54,437	226	1.16	(0.89, 1.51)
Mostly standing	18,303	87	1.00	
Using all of your major muscles	4125	21	0.99	(0.59, 1.65)

HR adjusted for age, sex, education levels, smoking status, drinking status, body mass index, long-term use of painkiller, long-term use of Chinese herbal medicine, hypertension, diabetes, hyperlipidemia, and physical activity

*ESRD* End-stage renal disease; *HR* Hazard ratio; *CI* Confidence interval

**Table 7:** Incident risk of chronic kidney disease, proteinuria, and lower eGFR for the participants with two visits by occupational sedentary status

Non-CKD to CKD	Mostly sitting				Often alternating sitting and standing				Mostly standing			Using all of your major muscles			
	*N*	*n* of CKD incidence	HR	(95% CI)	*N*	*n* of CKD incidence	HR	(95% CI)	*N*	*n* of CKD incidence	HR	*N*	*n* of CKD incidence	HR	(95% CI)
CKD incidence	104,749	4745	1.02	(0.95, 1.10)	48,790	2504	0.99	(0.92, 1.08)	16,597	1004	1.00	4230	252	0.92	(0.80, 1.06)

Non-CKD to proteinuria	Mostly sitting				Often alternating sitting and standing				Mostly standing			Using all of your major muscles			
	*N*	*n* of proteinuria incidence	HR	(95% CI)	*N*	*n* of proteinuria incidence	HR	(95% CI)	*N*	*n* of proteinuria incidence	HR	*N*	*n* of proteinuria incidence	HR	(95% CI)
Proteinuria incidence	104,749	3401	0.94	(0.86, 1.03)	48,790	1837	0.95	(0.87, 1.04)	16,597	714	1.00	4230	179	0.84	(0.71, 1.00)

Non-CKD to less than 60 mL/min/1.73 m^2^	Mostly sitting				Often alternating sitting and standing				Mostly standing			Using all of your major muscles			
	*N*	*n* of eGFR < 60 mL/min/1.73 m^2^ incidence	HR	(95% CI)	*N*	*n* of eGFR < 60 mL/min/1.73 m^2^ incidence	HR	(95% CI)	*N*	*n* of eGFR < 60 mL/min/1.73 m^2^ incidence	HR	*N*	*n* of eGFR < 60 mL/min/1.73 m^2^ incidence	HR	(95% CI)
Lower eGFR	104,749	1478	1.25	(1.09, 1.44)	48,790	713	1.10	(0.95, 1.27)	16,597	319	1.00	4230	77	1.11	(0.86, 1.45)

HR adjusted for age, sex, education levels, smoking status, drinking status, body mass index, long-term use of painkiller, long-term use of Chinese herbal medicine, hypertension, diabetes, hyperlipidemia, and physical activity

A total of 174,366 people with data on two visits and no CKD at the first visit. Of these people, a total of 8505 people developed CKD at the second visit; 6131 people had proteinuria at the second visit; 2587 had an eGFR < 60 mL/min/1.73 m^2^ at the second visit

*eGFR* Estimated glomerular filtration rate; *CKD* Chronic kidney disease; *HR* Hazard ratio; *CI* Confidence interval

**Table 8:** ESRD risk by occupational sedentary status, excluding participants who underwent dialysis or a had a kidney transplant within the first 2 years of follow-up or with CKD stage 5 (eGFR < 15 mL/min/1.73 m^2^)

	Mostly sitting			Often alternating sitting and standing			Mostly standing		Using all of your major muscles		
	*n* of ESRD	HR	(95% CI)	*n* of ESRD	HR	(95% CI)	*n* of ESRD	HR	*n* of ESRD	HR	(95% CI)
Exclude those ESRD within 2 years	1156	1.17	(1.01, 1.36)	613	1.03	(0.88, 1.21)	275	1	80	1.07	(0.82, 1.39)
Exclude CKD stage 5	1149	1.19	(1.03, 1.38)	621	1.07	(0.91, 1.25)	271	1	83	1.13	(0.87, 1.46)

Hazard ratios were adjusted for age, sex, education, smoking status, drinking status, body mass index, diabetes, hypertension, hyperlipidemia, long-term use of herbal medicine, long-term use of painkillers, and physical activity

*ESRD* End-stage renal disease; *CKD* Chronic kidney disease; *eGFR* Estimated glomerular filtration rate; *HR* Hazard ratio; *CI* Confidence interval

**Table 9:** Diabetes and overweight risk by occupational sedentary status

	Mostly sitting			Often alternating sitting and standing			Mostly standing		Using all of your major muscles		
	*n* of diseases	OR	(95% CI)	*n* of diseases	OR	(95% CI)	*n* of diseases	OR	*n* of diseases	OR	(95% CI)
Diabetes	12,908	1.26	(1.20, 1.32)	6230	1.11	(1.06, 1.17)	2573	1	684	0.99	(0.90, 1.09)
Overweight	69,786	1.15	(1.12, 1.18)	37,040	1.19	(1.16, 1.22)	14,658	1	4328	1.03	(0.99, 1.08)

Hazard ratios were adjusted for age, sex, education, smoking status, drinking status, body mass index, diabetes, hypertension, hyperlipidemia, long-term use of herbal medicine, long-term use of painkillers, and physical activity when appropriate

Diabetes was defined as having a history of diabetes or fasting blood glucose ≥ 126 mg/dL

Overweight was defined as BMI ≥ 25 (kg/m^2^), while normal weight was defined as 18.5 < BMI < 25 (kg/m^2^)

*OR* Odds ratio; *CI* Confidence interval; *DM* Diabetes mellitus; *BMI* Body mass index

## Code Availability

Code used for study analyses may be made available upon reasonable request to the authors.
